# Spatial Cognition in Adult and Aged Mice Exposed to High-Fat Diet

**DOI:** 10.1371/journal.pone.0140034

**Published:** 2015-10-08

**Authors:** James P. Kesby, Jane J. Kim, Miriam Scadeng, Gina Woods, Deborah M. Kado, Jerrold M. Olefsky, Dilip V. Jeste, Cristian L. Achim, Svetlana Semenova

**Affiliations:** 1 Department of Psychiatry, School of Medicine, University of California San Diego, La Jolla, California, United States of America; 2 Department of Pediatrics, School of Medicine, University of California San Diego, La Jolla, California, United States of America; 3 Department of Radiology, School of Medicine, University of California San Diego, La Jolla, California, United States of America; 4 Department of Family Medicine & Public Health and Internal Medicine, School of Medicine, University of California San Diego, La Jolla, California, United States of America; 5 Sam and Rose Stein Institute for Research on Aging, School of Medicine, University of California San Diego, La Jolla, California, United States of America; Texas Christian University, UNITED STATES

## Abstract

Aging is associated with a decline in multiple aspects of cognitive function, with spatial cognition being particularly sensitive to age-related decline. Environmental stressors, such as high-fat diet (HFD) exposure, that produce a diabetic phenotype and metabolic dysfunction may indirectly lead to exacerbated brain aging and promote the development of cognitive deficits. The present work investigated whether exposure to HFD exacerbates age-related cognitive deficits in adult versus aged mice. Adult (5 months old) and aged (15 months old) mice were exposed to control diet or HFD for three months prior to, and throughout, behavioral testing. Anxiety-like behavior in the light-dark box test, discrimination learning and memory in the novel object/place recognition tests, and spatial learning and memory in the Barnes maze test were assessed. HFD resulted in significant gains in body weight and fat mass content with adult mice gaining significantly more weight and adipose tissue due to HFD than aged mice. Weight gain was attributed to food calories sourced from fat, but not total calorie intake. HFD increased fasting insulin levels in all mice, but adult mice showed a greater increase relative to aged mice. Behaviorally, HFD increased anxiety-like behavior in adult but not aged mice without significantly affecting spatial cognition. In contrast, aged mice fed either control or HFD diet displayed deficits in novel place discrimination and spatial learning. Our results suggest that adult mice are more susceptible to the physiological and anxiety-like effects of HFD consumption than aged mice, while aged mice displayed deficits in spatial cognition regardless of dietary influence. We conclude that although HFD induces systemic metabolic dysfunction in both adult and aged mice, overall cognitive function was not adversely affected under the current experimental conditions.

## Introduction

A common, but not inevitable consequence of aging is a gradual decline in cognitive capabilities. Age-related cognitive deficits depend on specific cognitive domains, with some declining while others remain stable [1]. Given that not all older adults experience cognitive deficits, determining which factors can best facilitate maintaining cognitive abilities with age is of interest and considered among the most important aspects of successful aging [2,3]. Spatial cognition is especially sensitive to age-related cognitive decline [4] with age-related deficits reported in spatial learning and memory, and reversal learning [5,6].

Exposure to environmental stressors may be a driving factor in the differential susceptibility to age-related cognitive decline [7]. Furthermore, the escalating prevalence of obesity may exacerbate age-related cognitive decline. A systematic review of longitudinal population-based studies suggests that obesity in midlife is associated with lower cognitive functioning in later life [7]. However, the association between obesity in late life and cognitive abilities remains inconclusive [8]. Similarly, studies using rodent models indicate that the age of the animals when exposed to high-fat diet (HFD) is important for the development of cognitive impairment. For example, juvenile mice [9,10] and aged mice [11,12] appear more susceptible to HFD-induced impairments in learning and memory compared with adult mice.

The systemic effects of HFD exposure, such as increased fat mass, insulin resistance and general metabolic dysfunction have been identified as factors that may lead to impaired cognitive function [13]. For example, greater levels of visceral fat have been associated with impairments in the executive functioning of adolescents [14]. Furthermore, individuals with higher fasting insulin levels have a greater risk of developing Alzheimer’s disease [15]. Animal studies suggest that impairments in cognitive function after HFD exposure are associated with increased brain inflammation [16] and oxidative stress [11] that may be indirect consequences of systemic metabolic dysfunction. Importantly, the aging brain is considered to be particularly sensitive to these aspects of HFD exposure [11,13].

The goal of the present study was to characterize age-related impairments in spatial cognition and the impact of exposure to an environmental stressor, HFD. We hypothesized that spatial cognitive function would be impaired in aged mice compared to younger adult mice. Furthermore, exposure to HFD would induce systemic metabolic dysfunction and worsen or accelerate spatial cognitive impairments in aged and adult mice, respectively. To determine the systemic effects of HFD exposure we examined food consumption, fat mass content and fasting insulin/glucose levels. Behavioral testing included locomotor activity, anxiety-like behavior in the light-dark box test, object and place discrimination in the novel object/place recognition tasks and spatial learning, memory and reversal learning in the Barnes maze test. The results of the present studies demonstrated that although HFD exposure induced clear systemic metabolic dysfunction and aging led to impaired spatial cognition, HFD exposure did not further impair spatial cognitive function in adult or aged mice.

## Materials and Methods

### Animals

The present study used a total of 63 C57BL/6N male mice (Taconic Biosciences Inc., Taconic Farms Inc., Hudson, NY, USA) comprised of two age groups: adult (5 months old) and aged (15 months old, former breeders). Aged mice were 18–19 months old during behavioral testing to ensure that detected deficits in spatial learning were not attributed to loss of vision, smell and hearing. The mice were single-housed for the duration of the study in a humidity- and temperature-controlled animal facility on a 12 h/12 h reverse light/dark cycle (lights off at 7:00 AM) with *ad libitum* access to food and water. Behavioral testing was conducted during the dark phase of the light/dark cycle. All of the experiments were conducted in accordance with the guidelines of the American Association for the Accreditation of Laboratory Animal Care and National Research Council’s Guide for the Care and Use of Laboratory Animals and approved by the University of California San Diego Institutional Animal Care and Use Committee (Protocol S06137).

### Experimental timeline

Both adult (5 months old) and aged mice (15 months old) were separated into two groups (n = 15–16 per group) and placed on a control diet (CD; Teklad Diets 8604: 32% protein, 14% fat, 54% carbohydrates) or a HFD (Research Diets D12492: 20% protein, 60% fat, 20% carbohydrates). This HFD has been commonly used to induce obesity and produced insulin resistance [17], and reported to exacerbate cognitive decline in aged mice [16]. Food consumption was assessed by weighing daily food intake for three days after two months on the HFD and prior to behavioral testing (three months after beginning the HFD). Behavioral testing was completed in the following order: light-dark box test, locomotor activity, object/place recognition tests, and Barnes maze test. After Barnes maze completion, adult (9–10 months old) and aged (19–20 months old) mice were tested for fat mass content using magnetic resonance imaging, insulin sensitivity and glucose tolerance to confirm systemic physiological effects of HFD.

### Light-dark box test

The light-dark box test was conducted as previously described [18]. Three chambers were used for the light-dark box test (San Diego Instruments, San Diego, CA, USA). Each chamber consisted of two compartments: a dark compartment (16 × 21 × 33 cm) and a light compartment (26 × 21 × 33 cm) that were separated by a divider that left a 5 cm horizontal gap for the mouse to move from one compartment to the other. The light intensity in the middle of the light compartment was approximately 900 lux (as used in the Barnes maze, see below). In the dark compartment, the level of illumination was ~4 lux. Mice were placed in the dark compartment with their head facing away from the light compartment and the 5 min test was started. The total time spent in the light compartment and latency to the first transition to the light compartment (± 2.5 s) were recorded.

### Locomotor activity test

Locomotor activity was assessed in four open field arenas (60 x 60 cm) equipped with infra-red beams (Med Associates, St. Albans, VT, USA) to calculate total distance travelled. Mice were tested in the dark for a total of 60 min.

### Object and place recognition tests

Behavior was sequentially assessed in the novel object and novel place recognition tests using a 2-day protocol [19]. A Perspex box (63 x 42.5 x 22 cm), separated into three equal sized compartments was used [20]. Mice could move from one compartment to the next through centrally located openings (15 x 15 cm) that could be blocked with white removable dividing panels. Briefly, the novel object recognition test consisted of three 10-min phases: habituation, familiarization, and final test phases. For the familiarization phase, mice were confined to the middle compartment and two identical cups, either chrome with vertical bars (Galaxy pencil cup, Spectrum Diversified) or black with mesh grating (Nestable jumbo mesh pencil cup, WebOfficeMart), were placed (one per compartment) at one end of the side compartments. The dividers were removed and mice were given access to the entire arena and cups. For the test phase, one of the cups was replaced with an alternative/novel cup. The following day mice were tested in the novel place recognition test. The novel place recognition test was identical to the novel object recognition test but rather than replacing one cup with a novel cup in the test phase, one cup was moved to the opposite end of the side compartment. A scored interaction involved the nose oriented toward the object (no further than 1 cm). Mice were excluded from the analysis if they failed to interact with any of the objects during the familiarization session for less than 25 s in the familiarization session. Based on these criteria, a total of 6 mice were excluded from the novel object test (Adult mice: 1 control diet and 1 high-fat diet; Aged mice: 2 control diet and 2 high-fat diet) and 15 mice were excluded from the novel place test (Adult mice: 4 control diet and 5 high-fat diet; Aged mice: 2 control diet and 4 high-fat diet). The data were expressed as the following discrimination ratio of the duration of object exploration: (Novel—Sample) / Total. A positive discrimination ratio represents a greater level of interaction with the non-familiar object or place.

### Barnes maze test

The Barnes maze testing was conducted similar to that described previously [18]. The maze consisted of a white, acrylic, circular disc (90 cm diameter) that was elevated 90 cm above the floor, with 20 equally spaced holes (San Diego Instruments) with a black acrylic escape box (20 × 5 × 6 cm) placed under one of the holes. The maze was surrounded by four spatial cues at the height of the maze. Illumination in the center of the maze was approximately 900 lux. The maze was rotated 90 degrees each day to avoid the use of local cues on the maze by the mice.

#### Acquisition trials

Each mouse underwent 20 acquisition trials over 5 days, tested four times with an inter-trial interval of 10–15 min. Immediately prior to the first trial, all of the mice were individually placed into the escape tunnel for 1 min to avoid any neophobic responses. During testing, the mice were placed into a starting cylinder (10 cm diameter) in the center of the maze for 30 s. The cylinder was then removed, and the mouse was allowed to explore the arena to find the escape tunnel. The trial ended when the mouse entered the escape tunnel (i.e., when all four paws left the maze). When the mouse entered the escape tunnel, the entry was blocked, and the mouse was left in the tunnel for 1 min. If the mouse did not find or enter the escape tunnel within 3 min, then it was manually placed into the escape tunnel.

#### Probe trial

The 3-min probe trial was conducted on day 6 and was identical to the acquisition trials, with the exception that the escape tunnel was removed.

#### Memory retention

Two weeks after the probe test, the mice were tested for memory retention over four trials identical to the acquisition trials.

#### Reversal learning

For two days after the memory retention trials mice were tested for reversal learning. Each day consisted of four trials identical to the acquisition trials, but the location of the escape tunnel for each mouse was shifted 180°.

#### Behavioral measures

All behaviors were scored from video files by an experimenter who was blind to the experimental conditions and study hypotheses. The measures assessed were the latency to find the target hole, number of reference errors, number of working memory errors, and number of perseverative errors. Reference errors were defined as any incorrect hole inspection. Working memory errors were defined as searching the same hole twice within a trial when the revisit occurred after the inspection of other holes. Perseverative errors were defined as repeated searches of the same hole without searching another hole in between. Search strategy was also assessed in the acquisition, retention, and reversal trials. The search strategy was defined as one of three categories; random/mixed, serial, and spatial. A spatial strategy was defined as finding the target hole directly or after inspecting one of the adjacent holes first (thus having a maximum of one reference error). Random/mixed (< 74%) and serial (≥ 75%) strategy scores were all defined based on the percentage of errors that were made in a serial fashion. For an error to be defined as serial, this error had to be part of a minimum of three consecutive errors made in either direction around the maze without skipping a hole or changing direction. The percentage of the 4 trials per day that mice used each respective strategies were calculated. In the probe trial, the time spent by each mouse in the quadrant of the maze that contained the target hole was calculated.

### Fat mass magnetic resonance imaging

Analysis of fat distribution was studied using a Bruker 7 Tesla small animal MRI scanner (Bruker-Biospin, Ettlingen, Germany). Animals were anesthetized using 2% isoflurane. A T1 weighted imaging sequence was used to render the signal from fat high intensity, and non-fat tissues low intensity. Ninety-five 0.5 mm thick contiguous axial slices were acquired using a multi-slice, multi-echo sequence (Repetition time/Echo time = 1630.2 ms/10.5 ms), matrix 256 x 128, in-plane resolution 170 x 250 microns (varied slightly due to mouse size and hence field of view). Scans were averaged (3nex) to minimize blur from respiration. Imaging time was 10 minutes, 25 seconds. Using Amira software (FEI Hillsboro, Oregon USA), non-fat tissue was segmented from fat tissue using a threshold value of 2000–9600 for non-fat, and above 9600 for fat. Abdominal visceral and subcutaneous fat masses were separated by delineating the muscles of the abdominal wall. Scrotal fat was designated as abdominal fat.

### Fasting insulin and glucose testing

For fasting insulin/glucose measurements, mice were fasted for 8 hours prior to tail vein sampling. Whole blood glucose was measured using the OneTouch Ultra 2 glucometer. Plasma insulin was quantified using the Ultra Sensitive Mouse Insulin ELISA kit (ALPCO). The homeostasis model assessment of insulin resistance (HOMA-IR) was calculated using the following formula: [fasting blood glucose (mg/dl) × fasting insulin (μU/ml)] ÷ 405 [21].

### Statistical analyses

All of the analyses were performed with SPSS Statistics 19 (Chicago, IL, USA). All general and behavioral data were analyzed using analysis of variance (ANOVA), with *Age* and *Diet* as the between-subject factors. Repeated-measures ANOVAs were used when additional within-subject factors were present. When appropriate, *post hoc* comparisons were performed using Least Significant Difference (LSD) analyses. Results are expressed as mean ± SEM. Differences were considered statistically significant at *p* < 0.05.

## Results

### Body weight

At the beginning of the experiment, aged mice weighed more than adult mice (*F*_1,59_ = 162.2, *p* < 0.001). Over the course of the experiment, weight gain was dependent on both age and diet (Table 1). After 3 months on CD or HFD, significant main effects of *Age* (*F*_1,56_ = 34.9, *p* < 0.001) and *Diet* (*F*_1,56_ = 115.2, *p* < 0.001), and a significant interaction of *Age* x *Diet* (*F*_1,56_ = 95.0, *p* < 0.05) were observed. All groups were significantly different from one another with the adult mice on the CD weighing the least, followed by the aged mice on the CD, then the adult mice on the HFD with the aged mice on the HFD weighing the most. A similar profile was evident after 4.5 months, with significant main effects of *Age* (*F*_1,54_ = 17.6, *p* < 0.001) and *Diet* (*F*_1,54_ = 70.0, *p* < 0.001), in addition to a significant interaction of *Age* x *Diet* (*F*_1,54_ = 5.9, *p* < 0.05). However, at 4.5 months, there was no longer a significant difference in weight between adult and aged mice on the HFD. When weights were expressed as a percentage of baseline weight, adult mice gained 72% while aged mice gained 35%, indicating that the adult mice fed the HFD gained relatively more weight than their older counterparts.

**Table 1:** Effects of age and diet on weight (g) and weight gain (% of baseline).

	Adult-CD			Adult-HFD			Aged-CD			Aged-HFD		
	Mean	SEM	N	Mean	SEM	N	Mean	SEM	N	Mean	SEM	N
**Weight (g)**												
Baseline	29.8	0.5	16	29.5	0.5	16	39.9	1.1	15	40.3	1.1	16
3-months	34.2	0.8	16	48.3	0.9	16	43.1	1.4	15	52.1	1.1	13
4.5-months	36.6	1.1	16	50.8	1.0	16	45.3	1.6	15	53.1	1.5	11
**Weight (%)**												
3-months	114.5	1.5	16	163.6	2.6	16	107.9	1.6	15	132.8	2.9	13
4.5-months	122.3	2.3	16	172.3	3.8	16	113.4	2.2	15	134.6	5.4	11

CD, Control diet; HFD, High-fat diet.

### Food consumption and fat mass

**Fig 1:**
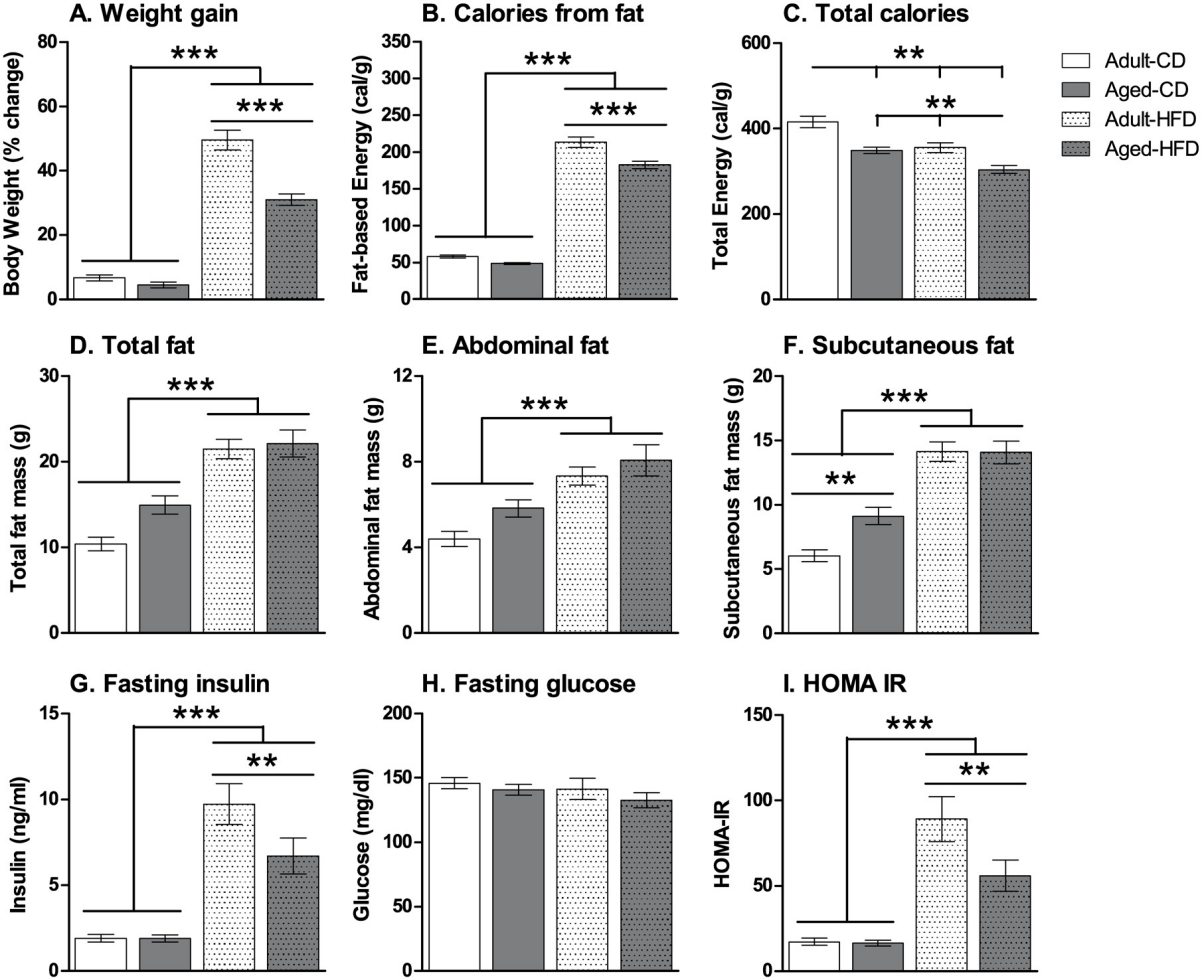
Weight gain, food consumption and physiological effects of aging and exposure to high-fat diet. *Top*: Weight gain (**A**), daily fat-based energy consumption per gram of bodyweight (**B**) and total energy consumption per gram of bodyweight (**C**) after 2 months on either the control diet (CD) or high-fat diet (HFD). All mice gained substantially more weight when consuming the HFD compared with the CD diet. However, adult mice gained a larger proportion of weight than aged mice when on the HFD (**A**). A similar profile was observed for the fat-based energy consumed per day (**B**) but not with total calorie consumption (**C**) suggesting that weight gain was driven by fat-based energy rather than total calorie consumption. *Middle*: Total fat mass (**D**), and the contributing amounts of abdominal fat (**E**) and subcutaneous fat (**F**) after 4.5 months on the CD or HFD. Both increased age and HFD exposure led to significantly greater total, subcutaneous and abdominal fat mass. However, aged mice on the CD had significantly higher subcutaneous fat mass than adult mice on the CD whereas no significant effect of age was observed in mice on the HFD. *Bottom*: Fasting insulin levels (**G**), fasting glucose levels (**H**) and Homeostasis Model Assessment of insulin response (HOMA-IR) (**I**) after 4.5 months on the CD or HFD. HFD exposure led to significantly increased levels of fasting insulin despite similar fasting glucose values, indicating significant HFD-induced insulin resistance as demonstrated by increased HOMA-IR levels. Adult mice on the HFD showed significantly greater fasting insulin levels and HOMA-IR values than aged mice on the HFD suggesting more severe metabolic effects of HFD consumption. The data are expressed as mean ± SEM. *p < 0.05, **p < 0.01, ***p < 0.001.

Food consumption and calorie intake were measured after 2 months on either the CD or the HFD. A similar profile of weight gain (Fig 1A) to that at three months was observed (see section 3.1). There were significant main effects of *Age* (*F*_1,59_ = 28.8, *p* < 0.001) and *Diet* (*F*_1,59_ = 322.0, *p* < 0.001), and a significant interaction of *Age* x *Diet* (*F*_1,59_ = 17.7, *p* < 0.001). A comparison of the profiles of the daily intake of fat-based calories versus total calories (per gram of bodyweight) suggested that at this time-point (after 2 months of HFD intake), fat content (Fig 1B) contributes more to weight gain than total calorie intake (Fig 1C). For example, for average calories sourced from fat there were significant main effects of *Age* (*F*_1,59_ = 19.2, *p* < 0.001) and *Diet* (*F*_1,59_ = 999.0, *p* < 0.001), and a significant interaction of *Age x Diet* (*F*_1,59_ = 5.5, *p* = 0.023). Mice on the HFD consumed significantly more fat-based calories than those on the CD. Adult mice on the HFD consumed significantly more fat-based calories than aged mice on the HFD (*p* < 0.001). Conversely, for total calorie intake there were significant main effects of *Age* (*F*_1,59_ = 29.6, *p* < 0.001) and *Diet* (*F*_1,59_ = 23.7, *p* < 0.001) with adult mice on the CD consuming the most calories and aged mice on the HFD consuming the least calories compared with the other groups (*p* < 0.01).

Fat mass was assessed using MRI after 4.5 months on either the CD or the HFD. For the total fat mass there were significant main effects of *Age* (*F*_1,53_ = 5.3, *p* < 0.05) and *Diet* (*F*_1,53_ = 65.7, *p* < 0.001) with both increased age and HFD consumption increasing fat mass (Fig 1D). A similar pattern was observed for abdominal fat mass with significant main effects of *Age* (*F*_1,53_ = 5.5, *p* < 0.05) and *Diet* (*F*_1,53_ = 31.6, *p* < 0.001) with both increased age and HFD consumption increasing fat mass (Fig 1E). For the subcutaneous fat mass there were significant main effects of *Age* (*F*_1,53_ = 4.7, *p* < 0.05) and *Diet* (*F*_1,53_ = 87.6, *p* < 0.001), and a significant interaction of *Age* x *Diet* (*F*_1,53_ = 5.1, *p* < 0.05). Both increased age and HFD consumption increased subcutaneous fat mass (Fig 1F). However, while aged mice on the CD had significantly higher subcutaneous fat mass than adult mice on the CD (*p* < 0.01), there was no significant difference between adult and aged mice on the HFD suggesting that adult mice gained relatively more subcutaneous fat due to the HFD than aged mice.

### Physiological effects

There were significant main effects of *Age* (*F*_1,43_ = 4.9, *p* < 0.05) and *Diet* (*F*_1,43_ = 85.5, *p* < 0.001), and a significant interaction of *Age x Diet* (*F*_1,43_ = 4.9, *p* < 0.05) for fasting insulin levels (Fig 1G). HFD significantly increased fasting insulin levels compared with the CD and this was effect was greater in adult mice compared with aged mice on the HFD (*p* < 0.001). Conversely, no significant effects of *Age* or *Diet* were observed on fasting glucose levels (Fig 1H). For the Homeostasis Model Assessment of insulin response (HOMA-IR) level (Fig 1I), which reflects insulin sensitivity, significant main effects of *Age* (*F*_1,43_ = 5.3, *p* < 0.05) and *Diet* (*F*_1,43_ = 57.6, *p* < 0.001), and a significant interaction of *Age x Diet* (*F*_1,43_ = 4.9, *p* < 0.05) were observed, with a similar pattern of results observed to those for fasting insulin levels.

### Anxiety-like behavior in the light-dark box test

**Fig 2:**
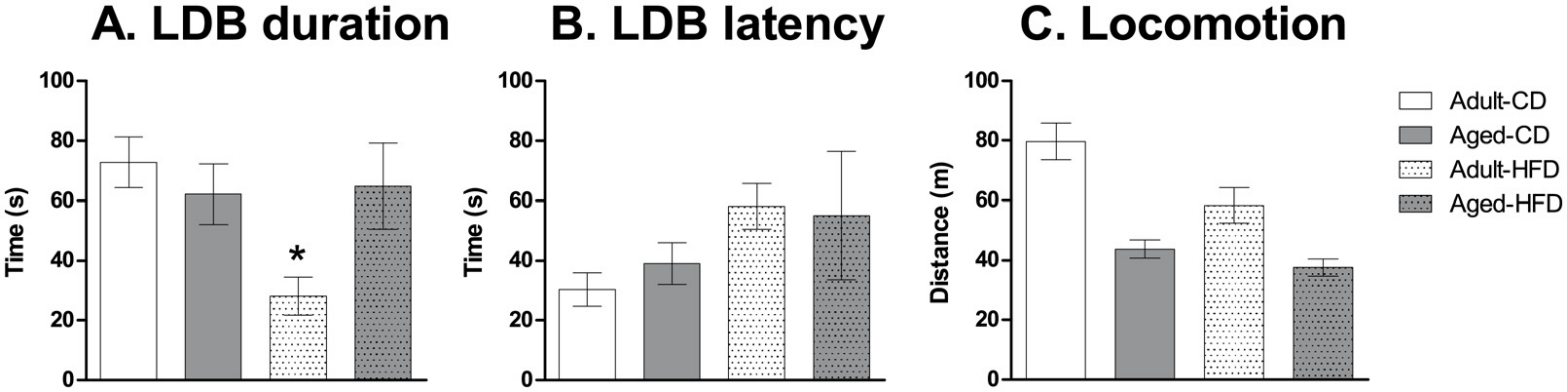
Effects of aging and exposure to high-fat diet on anxiety-like behavior and locomotor activity. Duration (s) in the light compartment (**A**) and latency to enter the light compartment (**B**) in the light-dark box test (LDB), and total distance travelled after 60 min in the open field (**C**) in adult and aged mice on a control diet (CD) or high-fat diet (HFD). Adult mice on the HFD spent less time in the light compartment than all other groups but no differences in the latency to enter the light compartment were observed. There were significant main effects of both age and diet on total distance travelled with aged mice travelling less than adult mice and mice on the HFD travelling less than mice on the CD diet. The data are expressed as mean ± SEM. *p < 0.05.

There was a significant main effect of *Diet* (*F*_1,56_ = 4.5, *p* < 0.05) and significant interaction of *Age* x *Diet* (*F*_1,56_ = 5.8, *p* < 0.05) observed on the duration spent in the light compartment of the light-dark box. Adult mice on HFD spent significantly less time in the light compartment than all other groups (Fig 2A). No significant main effects or interactions of *Age* or *Diet* were observed on the latency to enter the light compartment (Fig 2B).

### Locomotor activity

There were significant main effects of both *Age* (*F*_1,56_ = 33.0, *p* < 0.001) and *Diet* (*F*_1,56_ = 7.8, *p* < 0.01) with both increased age and HFD decreasing the total distance travelled in the open field (Fig 2C). For example, both aged mice and adult mice on the HFD showed decreased locomotor activity compared with adult mice and aged mice on CD, respectively. There was no significant interaction of *Age* x *Diet*.

### Novel object

**Fig 3:**
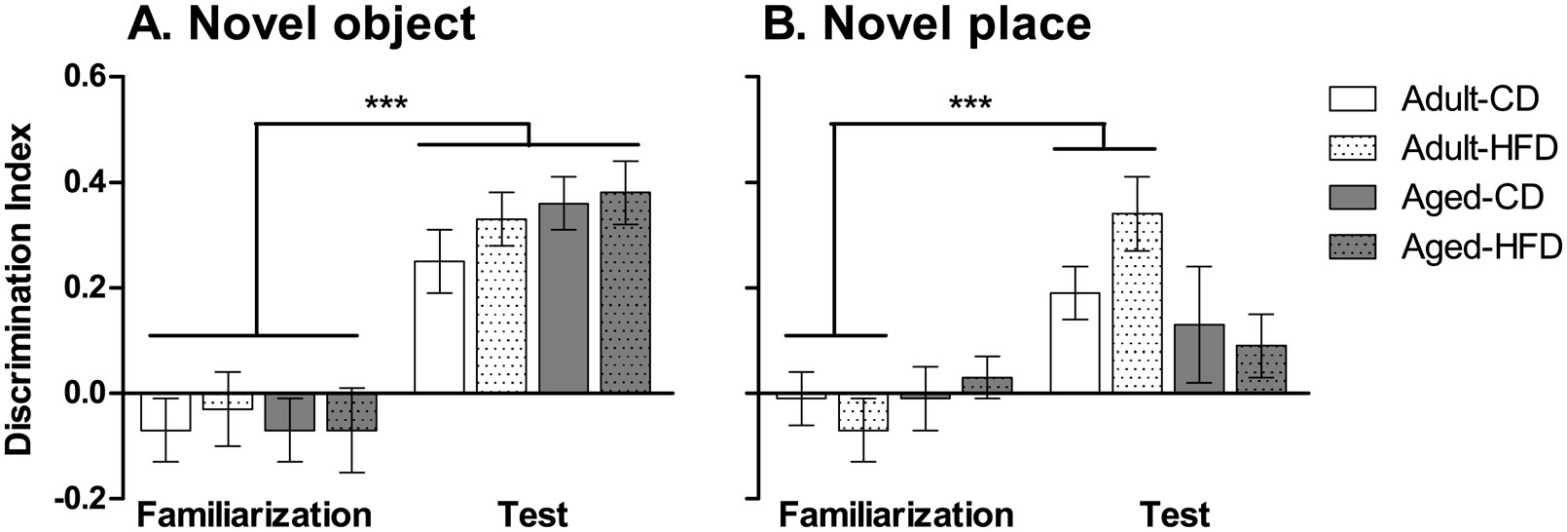
Effects of aging and exposure to high-fat diet on novel object and novel place recognition memory. Novel object (**A**) and novel place (**B**) recognition tasks in adult and aged mice on control diet (CD) or high-fat diet (HFD). Aged mice showed impaired novel place performance compared with adult mice, reflected by a similar discrimination index ([novel—familiar] / total) in the familiarization phase compared with the test phase of the task. No significant effects of HFD were evident. The data are expressed as mean ± SEM. ***p < 0.001.

There was a significant main effect of *Phase* on discrimination index (*F*_1,50_ = 83.8, *p* < 0.001) with mice showing a greater interaction bias between objects during the test phase compared with the familiarization phase (Fig 3A). No significant main effects or interactions of *Age* or *Diet* were observed in the total interaction times of either the familiarization or test phase (data not shown) or for any measures in novel object recognition.

### Novel place

There was a significant main effect of *Phase* on discrimination index (*F*_1,41_ = 17.0, *p* < 0.001) with mice showing a greater interaction bias between objects during the test phase compared with the familiarization phase (Fig 3B). A significant interaction of *Phase* x *Age* (*F*_1,41_ = 4.6, *p* < 0.05) was also observed. Adult mice displayed a significantly greater discrimination index in the test phase compared with the familiarization phase (*p* < 0.001), whereas aged mice did not. No significant effects of *Age* were observed in the total interaction times in either the familiarization or the test phase (data not shown). There were no significant effects of *Diet* for any measures in novel place recognition.

### Barnes maze acquisition trials

#### Latency

**Fig 4:**
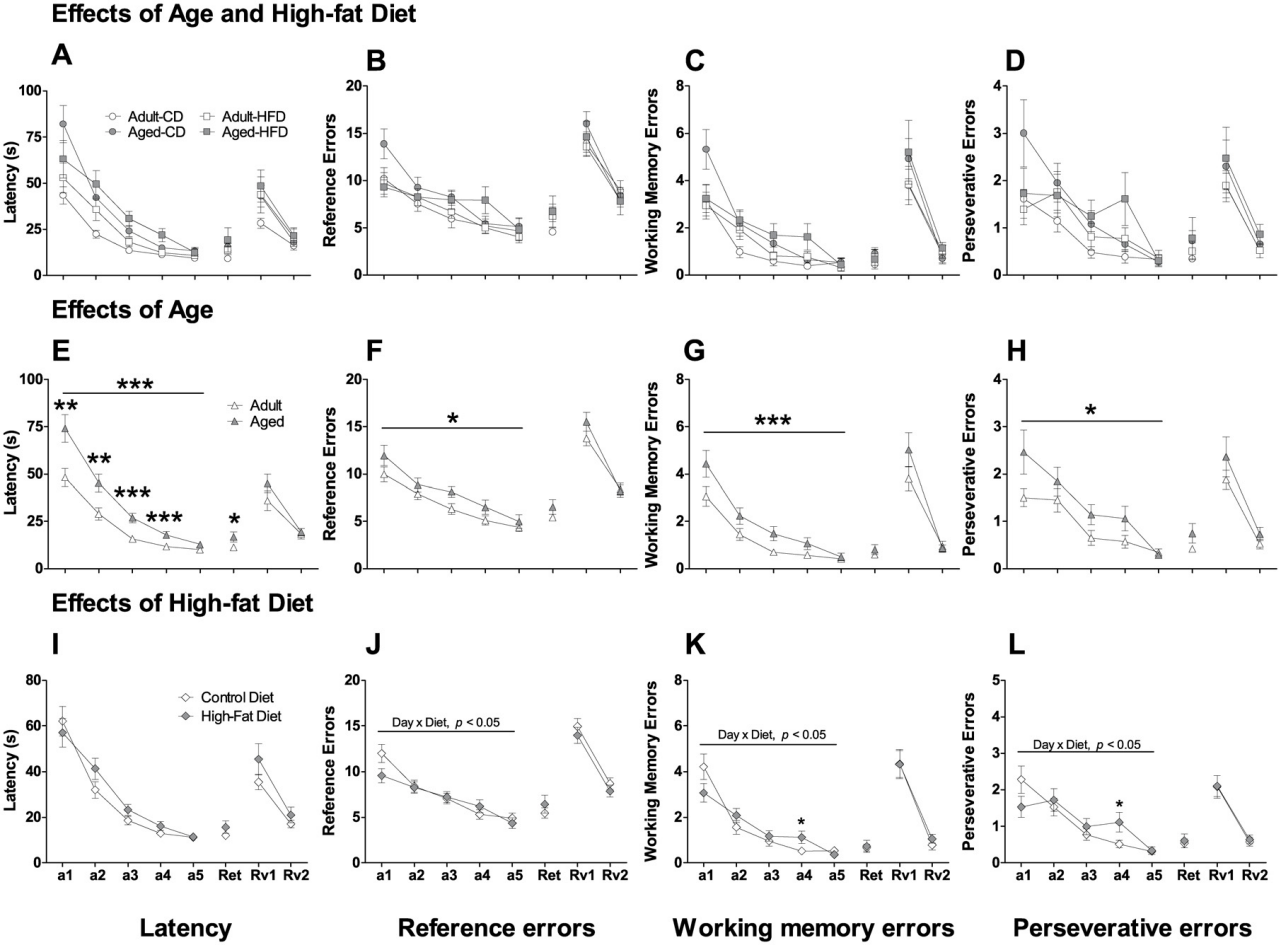
Effects of aging and exposure to high-fat diet on spatial cognition in the Barnes maze. Average latency (**A,E,I**), reference errors (**B,F,J**), working memory errors (**C,G,K**) and perseverative errors (**D,H,L**) by adult and aged mice on control diet (CD) and high-fat diet (HFD) on the Barnes maze acquisition days (a1-a5), retention day (Ret) and reversal learning days (Rv1-Rv2). In the top panels data for all groups are displayed whereas in the middle and bottom panels data are collapsed for age and diet, respectively. Aged mice performed worse than adult mice during the acquisition days as reflected by increases in latencies (**E**) and all errors (reference (**F**), working memory (**G**) and perseverative (**H**)). Mice on HFD tended to make more errors on day 4 of acquisition trials (**K,L**). The data are expressed as mean ± SEM. *p < 0.05, **p < 0.01, ***p < 0.001.

During task acquisition, significant main effects of *Day* (*F*_4,216_ = 86.4, *p* < 0.001) and *Trial* (*F*_3,162_ = 13.0, *p* < 0.001) were observed, with all mice exhibiting a reduction in the latency to find the target hole across the days of testing (Fig 4A). A significant main effect of *Age* (*F*_1,54_ = 19.7, *p* < 0.001) was also observed with aged mice taking significantly longer to locate the target hole than adult mice (Fig 4E). A significant interaction of *Day* x *Age* (*F*_4,216_ = 3.9, *p* < 0.05) was observed as aged mice took significantly longer than adult mice on days 1–4 (*p* < 0.01 for all) but not day 5 of training. There were no significant effects of *Diet* for latency during acquisition trials (Fig 4I).

#### Reference errors

During task acquisition, significant main effects of *Day* (*F*_4,216_ = 29.1, *p* < 0.001) and *Trial* (*F*_3,162_ = 3.0, *p* < 0.05) were observed, with all mice exhibiting a reduction in reference errors prior to finding the target hole across the days of testing (Fig 4B). A significant main effect of *Age* (*F*_1,54_ = 5.2, *p* < 0.05) was also observed with aged mice making more reference errors prior to locating the target hole than adult mice (Fig 4F). Although a significant interaction of *Day* x *Diet* was observed for reference errors (*F*_4,216_ = 2.5, *p* < 0.05), no meaningful group differences were observed (Fig 4J).

#### Working memory errors

During task acquisition, significant main effects of *Day* (*F*_4,216_ = 37.8, *p* < 0.001) and *Trial* (*F*_3,162_ = 5.9, *p* < 0.001) were observed, with all mice exhibiting a reduction in working memory errors prior to finding the target hole across the days of testing (Fig 4C). A significant main effect of *Age* (*F*_1,54_ = 14.0, *p* < 0.001) was also observed with aged mice making more working memory errors prior to locating the target hole than adult mice (Fig 4G). A significant interaction of *Day* x *Diet* was observed for working memory errors (*F*_4,216_ = 3.2, *p* < 0.05). Mice on the HFD made more working memory errors on Day 4 of acquisition compared with mice on the CD (*p* < 0.05) (Fig 4K).

#### Perseverative errors

During task acquisition, significant main effects of *Day* (*F*_4,216_ = 21.7, *p* < 0.001) and *Trial* (*F*_3,162_ = 4.4, *p* < 0.01) were observed, with all mice exhibiting a reduction in perseverative errors prior to finding the target hole across the days of testing (Fig 4D). A significant main effect of *Age* (*F*_1,54_ = 4.6, *p* < 0.05) was also observed with aged mice making more perseverative errors prior to locating the target hole than adult mice (Fig 4H). A significant interaction of *Day* x *Diet* was observed for perseverative errors (*F*_4,216_ = 3.5, *p* < 0.05). Mice on the HFD made more perseverative errors on Day 4 of acquisition compared with mice on the CD (*p* < 0.05) (Fig 4L).

#### Strategy use

**Fig 5:**
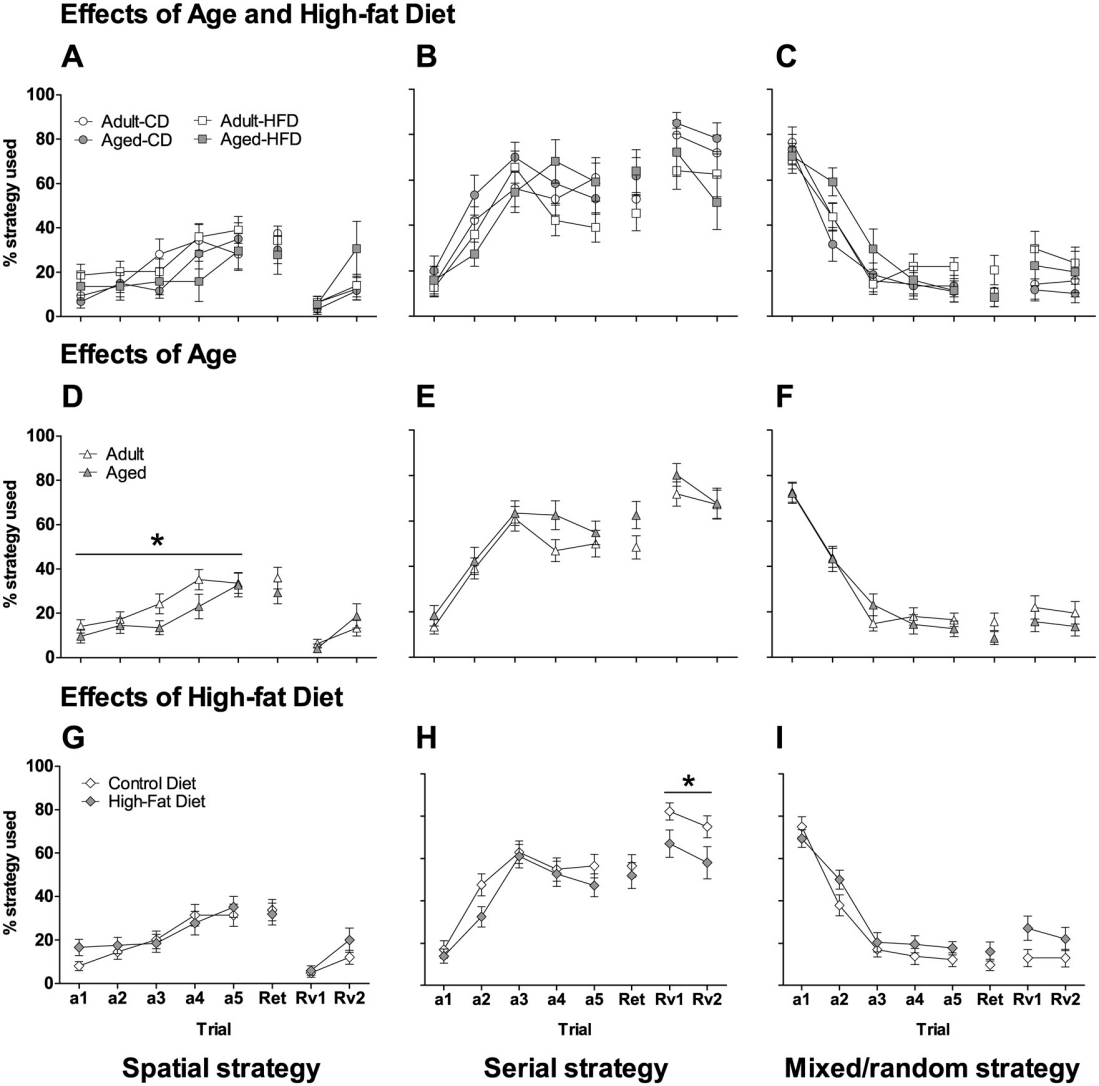
Effects of aging and exposure to high-fat diet on spatial strategy use in the Barnes maze. Spatial (**A,D,G**), serial (**B,E,H**) and mixed/random (**C,F,I**) strategy use by adult and aged mice on control diet (CD) and high-fat diet (HFD) on the Barnes maze acquisition days (a1-a5), retention day (Ret) and reversal learning days (Rv1-Rv2). In the top panels data for all groups are displayed, whereas in the middle and bottom panels data are collapsed for age and diet, respectively. Aged mice had significantly decreased spatial strategy use during the acquisition days compared with adult mice (**D**). Mice on the HFD used less serial strategy during the reversal learning days (**H**) and tended to use more mixed/random (**I**) compared with mice on CD. The data are expressed as mean ± SEM. *p < 0.05.

During task acquisition, a significant main effect of *Day* was observed for spatial (Fig 5A; *F*_4,216_ = 8.9, *p* < 0.001), serial (Fig 5B; *F*_4,216_ = 31.9, *p* < 0.001), and random/mixed (Fig 5C; *F*_4,216_ = 84.2, *p* < 0.001) strategies. However, a specific profile for each strategy used was observed across the days of testing. Spatial strategy frequency increased across the days of testing with the greatest use on days 4 and 5 (*p* < 0.05 vs. days 1–3). Serial strategy frequency also increased across the days of testing but the greatest use was on days 3–5 (*p* < 0.001 vs. days 1 and 2). Random/mixed strategy frequency decreased significantly from day1 to day 2 (*p* < 0.001) and again from day 2 to day 3 (*p* < 0.001) and plateaued on days 3–5. A significant main effect of *Age* (*F*_1,54_ = 4.8, *p* < 0.001) was observed for spatial strategy with aged mice using a spatial strategy less than adult mice (Fig 5D). The decrease in spatial strategy use observed in aged mice appeared to be due to marginal increases in both serial (Fig 5E) and mixed/random (Fig 5F) strategies. There was also a significant interaction of *Day* x *Age* x *Diet* (*F*_4,216_ = 3.4, *p* < 0.05) observed for spatial strategy used. However, no meaningful group differences were observed (Fig 5A).

### Barnes maze probe trial

There was a significant difference in the duration of time spent in each quadrant of the Barnes maze (significant main effect of *Quadrant* (*F*_3,162_ = 64.9, *p* < 0.001), data not shown). Mice spent significantly more time in the target quadrant than all others (*p* < 0.001) and significantly less time in the opposite quadrant than all others (*p* < 0.05). However, there were no significant effects or interactions of *Age* or *Diet* on time spent in any quadrant during the probe trial.

### Barnes maze retention trials

There was a significant main effect of *Age* (*F*_1,52_ = 4.7, *p* < 0.05) for latency, with aged mice taking longer than adult mice to locate the target hole (Fig 4E). There were no significant effects of *Age* on reference, working memory, and perseverative errors, or strategy use in the retention trials. There were no significant effects of *Diet* on any measure during the retention trials.

### Barnes maze reversal trials

There were no significant effects of *Age* on latency, errors including reference, working memory, and perseverative errors, or strategy use in the reversal trials (Figs 4 and 5). In the Barnes maze reversal trials a significant main effect of *Diet* (*F*_1,52_ = 5.6, *p* < 0.05) was observed for serial strategy use. Compared with mice on CD, mice on HFD used less serial strategies (Fig 5H) with a concomitant increase in the use of random/mixed strategies (Fig 5I), although this effect did not reach significance. There were no significant effects of *Diet* on any other measures during the reversal trials.

## Discussion

The results of the present study showed that exposure to the HFD resulted in metabolic dysfunction as reflected by significant weight gain, fat mass gain, increased fasting insulin levels and insulin resistance. Surprisingly, adult mice appeared to be affected by HFD exposure to a greater extent than aged mice, showing comparatively higher fasting insulin levels, greater gains in subcutaneous fat mass and larger weight gain. Exposure to HFD also increased anxiety-like behavior in adult mice only. Consistent with our hypothesis, aged mice showed impairments in spatial learning (Barnes maze acquisition) and spatial recognition memory (novel place discrimination). However, contrary to our hypothesis, HFD exposure did not significantly contribute to any age-related impairments in spatial cognition in either adult or aged mice.

Age-dependent alterations in anxiety-like behavior were observed in mice on HFD. Studies in adult human subjects have reported that diets high in processed food (more similar to ‘western diets’) are associated with increased anxiety [22]. In the light-dark box test, adult, but not aged, mice on HFD spent less time in the light compartment compared with the other groups, suggesting increased anxiety-like behavior. This is consistent with other work showing increased anxiety-like behavior in mice exposed to HFD during adulthood [23], but not at puberty [24]. Our findings suggest that there may be a susceptible window in adulthood for HFD-induced increases in anxiety-like behavior.

Age-related impairments in spatial learning in the Barnes maze or Morris water maze are commonly reported in rodents [25,26]. The hippocampus, in particular, is a critical region involved in both age-related cognitive impairments and spatial learning/memory in both humans and rodents [27,28]. In our study, aged mice made more reference, working memory, and perseverative errors during the acquisition trials compared with adult mice. However, both age groups exhibited similar performance by the end of the acquisition trials indicating that aged mice were as capable of learning the task as adult mice but were slower to reach a similar level of competence. This finding was consistent with similar time spent in the target quadrant during the probe trial. Age-related impairments were limited to spatial learning (Barnes maze acquisition) and place discrimination (novel place test) and not object discrimination (novel object test) or executive functioning (Barnes maze reversal). Taken together, our findings, based upon testing that is thought to be representative of hippocampal function, suggest that the hippocampus is more sensitive to age-related impairments than the cortical regions implicated in executive functions [29] and novel object discrimination [30].

Mice on HFD showed relatively unimpaired cognitive performance when compared to mice on CD. Our results are consistent with other studies showing no cognitive impairments in mice and rats exposed to HFD and tested for spatial learning in the Morris water maze at various ages [31–33]. However, other studies using the T-maze to assess spatial learning and memory retention have observed HFD-induced cognitive impairments [11,16]. Thus, certain aspects of spatial cognition may be differentially sensitive to impairments by HFD.

The physiological effects of HFD exposure and associated obesity, such as increased fasting insulin and insulin resistance, are well described in both rodents [34] and humans [35]. For example, HFD and obesity have been shown to result in the accumulation of immune cells such as activated macrophages in adipose and liver tissue causing the secretion of pro-inflammatory cytokines that can directly impair insulin sensitivity in insulin target cells [17]. Although aging alone had only subtle effects on systemic physiology (increased subcutaneous fat mass), age played an important role in the impact of HFD exposure. For example, HFD-induced increases in fasting insulin levels and HOMA-IR were more severe in adult mice than aged mice. These more drastic metabolic effects in adult versus ages mice may in turn contribute to the larger gains in subcutaneous fat mass as well as increases in anxiety-like behavior observed only in this group. Overall however, these data suggest that impaired insulin sensitivity, weight gain and fat mass content do not directly impact spatial cognition or its decline with age under the current experimental conditions. However, age-dependent effects on anxiety-like behavior do suggest that under certain conditions (i.e. time of life) systemic metabolic dysfunction may alter brain function.

## Conclusions

Age-related impairments in spatial cognition in both humans and mice occur, but the underlying etiology is not well understood. Our study in adult and aged mice confirms aged-related spatial cognitive deficits, but does not support a causative role of HFD. The HFD did induce expected changes in weight gain and systemic metabolic dysfunction in both adult and aged mice. The effects of HFD also differed by age, with the adult mice experiencing the greatest changes. Our finding suggest that anxiety-like behavior may be more susceptible to HFD exposure during midlife rather than later in life. This effect may be attributable to increased susceptibility to the physiological effects of HFD exposure in adult mice. Taken together, these results suggest that the time of greatest intervention may be in mid-life. Future research as to whether HFD may induce an acceleration of deficits in other aspects of cognition is warranted.

## References

[1] Burke SN, Barnes CA (2006) Neural plasticity in the ageing brain. Nature Rev Neurosci 7: 30–40. 16371948 10.1038/nrn1809

[2] Depp CA, Glatt SJ, Jeste DV (2007) Recent advances in research on successful or healthy aging. Curr Psychiatry Rep 9: 7–13. 17257507 10.1007/s11920-007-0003-0

[3] Jeste DV, Depp CA, Vahia IV (2010) Successful cognitive and emotional aging. World Psychiatry 9: 78–84. 20671889 10.1002/j.2051-5545.2010.tb00277.x PMC2912035

[4] Klencklen G, Despres O, Dufour A (2012) What do we know about aging and spatial cognition? Reviews and perspectives. Ageing Res Rev 11: 123–135. 22085884 10.1016/j.arr.2011.10.001

[5] Schoenfeld R, Foreman N, Leplow B (2014) Ageing and spatial reversal learning in humans: Findings from a virtual water maze. Behav Brain Res 270: 47–55. 10.1016/j.bbr.2014.04.036 24815214

[6] Yamamoto N, DeGirolamo GJ (2012) Differential effects of aging on spatial learning through exploratory navigation and map reading. Front Aging Neurosci 4: 14 10.3389/fnagi.2012.00014 22701423 PMC3372958

[7] Dahl AK, Hassing LB (2013) Obesity and Cognitive Aging. Epidemiol Rev 35: 22–32. 10.1093/epirev/mxs002 23258415

[8] Aslan AK, Starr JM, Paiite A, Deary I (2014) Cognitive consequences of overweight and obesity in the ninth decade of life? Age Ageing 44: 59–65. 10.1093/ageing/afu108 25249169

[9] Boitard C, Cavaroc A, Sauvant J, Aubert A, Castanon N, et al (2014) Impairment of hippocampal-dependent memory induced by juvenile high-fat diet intake is associated with enhanced hippocampal inflammation in rats. Brain Behav Immun 40: 9–17. 10.1016/j.bbi.2014.03.005 24662056

[10] Valladolid-Acebes I, Fole A, Martin M, Morales L, Cano MV, et al (2013) Spatial memory impairment and changes in hippocampal morphology are triggered by high-fat diets in adolescent mice. Is there a role of leptin? Neurobiol Learn Mem 106: 18–25. 10.1016/j.nlm.2013.06.012 23820496

[11] Morrison CD, Pistell PJ, Ingram DK, Johnson WD, Liu Y, et al (2010) High fat diet increases hippocampal oxidative stress and cognitive impairment in aged mice: implications for decreased Nrf2 signaling. J Neurochem 114: 1581–1589. 10.1111/j.1471-4159.2010.06865.x 20557430 PMC2945419

[12] Tucsek Z, Toth P, Tarantini S, Sosnowska D, Gautam T, et al (2014) Aging exacerbates obesity-induced cerebromicrovascular rarefaction, neurovascular uncoupling, and cognitive decline in mice. J Gerontol A Biol Sci Med Sci 69: 1339–1352. 10.1093/gerona/glu080 24895269 PMC4204615

[13] Uranga RM, Bruce-Keller AJ, Morrison CD, Fernandez-Kim SO, Ebenezer PJ, et al (2010) Intersection between metabolic dysfunction, high fat diet consumption, and brain aging. J Neurochem 114: 344–361. 10.1111/j.1471-4159.2010.06803.x 20477933 PMC2910139

[14] Schwartz DH, Leonard G, Perron M, Richer L, Syme C, et al (2013) Visceral fat is associated with lower executive functioning in adolescents. Int J Obes (Lond) 37: 1336–1343. 23797144 10.1038/ijo.2013.104 PMC5061567

[15] Luchsinger JA, Tang MX, Shea S, Mayeux R (2004) Hyperinsulinemia and risk of Alzheimer disease. Neurology 63: 1187–1192. 15477536 10.1212/01.wnl.0000140292.04932.87

[16] Pistell PJ, Morrison CD, Gupta S, Knight AG, Keller JN, et al (2010) Cognitive impairment following high fat diet consumption is associated with brain inflammation. J Neuroimmunol 219: 25–32. 10.1016/j.jneuroim.2009.11.010 20004026 PMC2823983

[17] Xu J, Morinaga H, Oh D, Li P, Chen A, et al (2012) GPR105 ablation prevents inflammation and improves insulin sensitivity in mice with diet-induced obesity. J Immunol 189: 1992–1999. 10.4049/jimmunol.1103207 22778393 PMC3411902

[18] Kesby JP, Markou A, Semenova S, TMARC (2015) Cognitive deficits associated with combined HIV gp120 expression and chronic methamphetamine exposure in mice. Eur Neuropsychopharm 25: 141–150. 10.1016/j.euroneuro.2014.07.014 PMC4289653 25476577

[19] Barnes SA, Pinto-Duarte A, Kappe A, Zembrzycki A, Metzler A, et al (2015) Disruption of mGluR5 in parvalbumin-positive interneurons induces core features of neurodevelopmental disorders. Mol Psychiat: In press. 10.1038/mp.2015.113 PMC4583365 26260494

[20] Nadler JJ, Moy SS, Dold G, Trang D, Simmons N, et al (2004) Automated apparatus for quantitation of social approach behaviors in mice. Genes Brain Behav 3: 303–314. 15344923 10.1111/j.1601-183X.2004.00071.x

[21] Matthews DR, Hosker JP, Rudenski AS, Naylor BA, Treacher DF, et al (1985) Homeostasis model assessment: insulin resistance and beta-cell function from fasting plasma glucose and insulin concentrations in man. Diabetologia 28: 412–419. 3899825 10.1007/BF00280883

[22] Bakhtiyari M, Ehrampoush E, Enayati N, Joodi G, Sadr S, et al (2013) Anxiety as a consequence of modern dietary pattern in adults in Tehran-Iran. Eat Behav 14: 107–112. 10.1016/j.eatbeh.2012.12.007 23557804

[23] Souza CG, Moreira JD, Siqueira IR, Pereira AG, Rieger DK, et al (2007) Highly palatable diet consumption increases protein oxidation in rat frontal cortex and anxiety-like behavior. Life Sci 81: 198–203. 17574275 10.1016/j.lfs.2007.05.001

[24] Finger BC, Dinan TG, Cryan JF (2011) High-fat diet selectively protects against the effects of chronic social stress in the mouse. Neuroscience 192: 351–360. 10.1016/j.neuroscience.2011.06.072 21742017

[25] Bach ME, Barad M, Son H, Zhuo M, Lu YF, et al (1999) Age-related defects in spatial memory are correlated with defects in the late phase of hippocampal long-term potentiation in vitro and are attenuated by drugs that enhance the cAMP signaling pathway. PNAS 96: 5280–5285. 10220457 10.1073/pnas.96.9.5280 PMC21855

[26] Gerstein H, Lindstrom MJ, Burger C (2013) Gene delivery of Homer1c rescues spatial learning in a rodent model of cognitive aging. Neurobiol Aging 34: 1963–1970. 10.1016/j.neurobiolaging.2013.02.006 23523268 PMC3651797

[27] Haberman RP, Colantuoni C, Koh MT, Gallagher M (2013) Behaviorally activated mRNA expression profiles produce signatures of learning and enhanced inhibition in aged rats with preserved memory. Plos One 8: e83674 10.1371/journal.pone.0083674 24349543 PMC3862806

[28] Yassa MA, Lacy JW, Stark SM, Albert MS, Gallagher M, et al (2011) Pattern separation deficits associated with increased hippocampal CA3 and dentate gyrus activity in nondemented older adults. Hippocampus 21: 968–979. 10.1002/hipo.20808 20865732 PMC3010452

[29] Kesby JP, Heaton RK, Young JW, Umlauf A, Woods SP, et al (2015) Methamphetamine Exposure Combined with HIV–1 Disease or gp120 Expression: Comparison of Learning and Executive Functions in Humans and Mice. Neuropsychopharmacology 40: 1899–1909. 10.1038/npp.2015.39 25652249 PMC4839513

[30] Barker GRI, Warburton EC (2011) When Is the Hippocampus Involved in Recognition Memory? J Neurosci 31: 10721–10731. 10.1523/JNEUROSCI.6413-10.2011 21775615 PMC6622630

[31] McNeilly AD, Williamson R, Sutherland C, Balfour DJK, Stewart CA (2011) High fat feeding promotes simultaneous decline in insulin sensitivity and cognitive performance in a delayed matching and non-matching to position task. Behav Brain Res 217: 134–141. 10.1016/j.bbr.2010.10.017 20974195

[32] Mielke JG, Nicolitch K, Avellaneda V, Earlam K, Ahuja T, et al (2006) Longitudinal study of the effects of a high-fat diet on glucose regulation, hippocampal function, and cerebral insulin sensitivity in C57BL/6 mice. Behav Brain Res 175: 374–382. 17081630 10.1016/j.bbr.2006.09.010

[33] Pancani T, Anderson KL, Brewer LD, Kadish I, DeMoll C, et al (2013) Effect of high-fat diet on metabolic indices, cognition, and neuronal physiology in aging F344 rats. Neurobiol Aging 34: 1977–1987. 10.1016/j.neurobiolaging.2013.02.019 23545425 PMC3651766

[34] Xu CX, Wang C, Zhang ZM, Jaeger CD, Krager SL, et al (2015) Aryl hydrocarbon receptor deficiency protects mice from diet-induced adiposity and metabolic disorders through increased energy expenditure. Int J Obes (Lond) In press. 10.1038/ijo.2015.63 PMC4526411 25907315

[35] Ouchi N, Parker JL, Lugus JJ, Walsh K (2011) Adipokines in inflammation and metabolic disease. Nat Rev Immunol 11: 85–97. 10.1038/nri2921 21252989 PMC3518031

